# Minute-Scale Sleep-Onset HRV as a Candidate Risk-Stratification Biomarker for Diabetic Retinopathy: An Exploratory Pilot Study

**DOI:** 10.3390/diagnostics16203301

**Published:** 2026-10-11

**Authors:** Yi-Ting Cheng, Yu-Cheng Lin, Yu-Ting Ho, Terry B. J. Kuo, Cheryl C. H. Yang

**Affiliations:** 1 Institute of Brain Science, National Yang Ming Chiao Tung University, No. 155, Sec. 2, Li-Nong St., Beitou, Taipei 11221, Taiwan; yyttcc.md14@nycu.edu.tw (Y.-T.C.); simyuyu@gmail.com (Y.-C.L.);; 2 Sleep Research Center, National Yang Ming Chiao Tung University, Taipei 11221, Taiwan; 3 Tsaotun Psychiatric Center, Ministry of Health and Welfare, Nantou 115204, Taiwan; 4 Department of Education and Research, Taipei City Hospital, Taipei 103212, Taiwan; 5 Brain Research Center, National Yang Ming Chiao Tung University, Taipei 11221, Taiwan

**Keywords:** heart rate variability, minute-scale HRV, SDANN, diabetic retinopathy, autonomic neuropathy, sleep-onset transition, screening biomarker, risk stratification, wearable screening

## Abstract

**Background**: Diabetic retinopathy (DR) is the most common microvascular complication of type 2 diabetes mellitus (T2DM), yet DR screening coverage remains inadequate worldwide. Diabetic autonomic neuropathy typically precedes DR, and heart rate variability (HRV) provides a non-invasive autonomic measure; however, whether HRV can screen for DR remains unexplored. **Methods**: This pilot study investigated whether sleep-onset minute-scale HRV indices—SDANN1 (standard deviation of 1-min segment means of normal-to-normal intervals) and SDANN2 (2-min segments)—serve as candidate screening biomarkers. Using a public 24-h Holter ECG dataset of T2DM inpatients (*N* = 39; 11 DR-positive, 28 DR-negative), sleep-onset time-points from cardiopulmonary coupling analysis were used to extract 30 min of post-onset ECG. **Results**: SDANN1 was significantly lower in the DR group (median 23.6 vs. 44.8 ms, *p* = 0.028, d = 0.74), with an area under the receiver operating characteristic curve of 0.731 (95% CI: 0.530–0.901). At the optimal threshold of ≤ 25 ms, the odds ratio was 8.1 (95% CI: 1.7–38.4), with DR prevalence of 58% in the high-risk versus 12.5% in the low-risk group. Among 12 candidates, only the SDANN family reached significance. **Conclusions**: sleep-onset minute-scale HRV shows promise as a candidate risk-stratification biomarker for DR; prospective longitudinal validation in larger, sex-balanced cohorts is required before any screening application.

## 1. Introduction

### 1.1. The Screening Dilemma in Diabetic Retinopathy

Diabetic retinopathy (DR) is the most common microvascular complication of type 2 diabetes mellitus (T2DM). Globally, approximately one-third of patients with diabetes will develop DR [1]. DR is the leading cause of blindness in the working-age population, yet it is entirely asymptomatic in the early stages and largely preventable through timely detection and treatment [2].

The current DR screening pathway relies largely on retinal examination or retinal imaging, which requires appropriate equipment, trained personnel, and healthcare resources. These resource requirements, together with variability in screening infrastructure, remain important barriers to achieving adequate DR screening coverage globally [3]. Reported screening uptake falls well short of recommended levels in many settings [4]. Although AI-assisted fundus image recognition (e.g., the U.S. Food and Drug Administration [FDA]-approved IDx-DR) has achieved breakthroughs in automated screening with area under the curve (AUC) values exceeding 0.95 [5,6], this approach still requires fundus photography equipment, limiting its accessibility in remote areas and primary care.

With the widespread adoption of wearable devices, smart wristbands now routinely provide long-duration heart rate variability (HRV) monitoring, with particularly high signal quality and clinical feasibility during continuous sleep recording. Developing an HRV-based DR screening adjunct that does not rely on fundus imaging and can be deployed via wearables is therefore of clinical urgency and practical feasibility.

### 1.2. The Link Between Diabetic Autonomic Neuropathy and Retinopathy

Diabetes induces oxidative stress through chronic hyperglycemia, progressively damaging the microvasculature and nervous system [7,8]. In this pathological process, diabetic autonomic neuropathy (DAN) can precede retinopathy. Multiple longitudinal studies support this temporal relationship: the EURODIAB Prospective Complications Study demonstrated that diabetic neuropathy and retinopathy share common modifiable cardiovascular risk factors, supporting converging pathological pathways [9]; Bitirgen et al. demonstrated using corneal confocal microscopy that corneal nerve fibre damage precedes diabetic retinopathy in type 2 diabetes patients, supporting the temporal hypothesis that nerve injury precedes retinopathy [10]. DAN and DR share the same pathological mechanisms—microvascular damage and oxidative stress—but DAN occurs earlier on the timeline, implying that early detection of DAN may serve as an early-warning signal for DR. Complementing such physiological markers, molecular biomarker-discovery efforts in DR—for example, mitochondrial unfolded-protein-response gene signatures—illustrate the breadth of current approaches to earlier DR detection [11].

HRV is a widely used non-invasive method for assessing autonomic function [12]. Reduced HRV is broadly accepted as a hallmark of DAN [13,14]. However, the existing HRV–diabetes literature has three key gaps: first, most HRV studies exclude patients with diabetes, overlooking clinically meaningful gradations within the “low” range; second, only short 5-min recordings are typically used, which cannot capture slower autonomic dynamics; third, HRV has been used only for DAN diagnosis, with few studies leveraging the temporal lead of DAN over DR to use HRV as a predictive tool for DR.

The few studies that have examined HRV–DR associations illustrate these limitations. Kaze et al. derived HRV measures from ECGs recorded over 10 s in 5810 participants and found that low SDNN was associated with incident vision-threatening DR (adjusted hazard ratio 1.32, 95% CI 1.03–1.71) [15]. However, ultra-short 10-s recordings severely constrain the range of HRV features extractable and preclude conventional frequency-domain or minute-scale analysis. Khandoker et al. reported generally reduced HRV in 169 patients with T2DM, 106 of whom had retinopathy, yet individual time-domain and frequency-domain parameters yielded odds ratios near unity, with only the LF/HF ratio showing moderate associations (OR 1.33–4.92) [16]. Galicka-Latała et al. reported significantly lower frequency-domain HRV, particularly HF power, in patients with type 1 diabetes and retinopathy during sleep [17]. However, their analyses were limited to group-level comparisons and did not evaluate individual-level diagnostic discrimination. Critically, none of these studies reported AUC, sensitivity, or specificity for standalone HRV-based DR screening, none examined minute-scale indices such as SDANN, and none targeted the sleep-onset transition as the observation window. These gaps motivate the present study.

### 1.3. The Sleep-Onset Transition: A Natural Autonomic Regulatory Challenge

The sleep-onset transition is the time window during which the autonomic nervous system switches from sympathetic to parasympathetic dominance. During this transition, heart rate fluctuates dynamically on a minute scale; when autonomic regulation is impaired in DAN, these fluctuations may be attenuated. Thus, HRV changes during the sleep-onset transition may serve as an observation window for evaluating autonomic function. Classical physiological studies substantiate this transition: during non-rapid-eye-movement sleep, autonomic balance shifts from sympathetic toward parasympathetic dominance, with an abrupt change at sleep onset [18]; heart rate and blood pressure fall substantially across the sleep-onset period, accompanied by baroreflex resetting [19]; and time–frequency analysis of heart rate variability shows that the wake–sleep transition involves a step-change resetting of autonomic function that begins with a decrease in very slow heart-rate oscillations minutes before sleep onset [20]. In diabetic autonomic neuropathy, the nocturnal increase in vagal activity and the day–night variation in sympathovagal balance are progressively blunted [21]. Together, these observations position the sleep-onset transition as the window in which impaired autonomic regulation should be most readily observable.

Cheng W et al. found that sleep-period HRV reflects autonomic dysfunction and metabolic status better than waking-period HRV [22]. Building on this, we further hypothesize that HRV during the sleep-onset transition—rather than during stable sleep—is the critical window for discriminating patients with DR.

### 1.4. SDANN: An Under-Utilized Minute-Scale Index

The standard deviation of the averages of normal-to-normal (NN) intervals (SDANN) was used in early 24-h HRV research by Bigger et al. in 1988 [23] and was subsequently included among the four standard time-domain measures recommended for long-term HRV analysis by the 1996 Task Force of the European Society of Cardiology and the North American Society of Pacing and Electrophysiology [12]. Traditional SDANN uses 5-min segments and was designed for 24-h Holter recordings. With the increasing use of wearable devices, RMSSD has emerged as a commonly reported HRV metric, whereas SDANN, which is primarily intended for long-term recordings, is less applicable to short-duration assessments [24,25].

Paradoxically, SDANN may be better suited to wearable-grade signals than RMSSD. Wearable devices inevitably introduce motion artifacts and occasional missed or spurious R-peak detections. RMSSD, computed from consecutive beat-to-beat differences, is highly sensitive to even a single artifact beat, which can drastically inflate or deflate the metric. SDANN, by contrast, first averages RR intervals within each segment and then computes the standard deviation of these segment means; this two-stage architecture naturally smooths out sporadic artifacts, conferring inherent noise robustness. This structural advantage makes SDANN a promising candidate for clinical deployment on consumer wearable platforms where signal quality cannot be guaranteed.

Notably, wearable devices have demonstrated clinically relevant physiological monitoring capabilities. An Apple Watch feature has received FDA authorization for notifying users of irregular rhythms suggestive of atrial fibrillation [26], and its performance has been evaluated in a large-scale pragmatic study [27]. A separate Apple Watch feature has also received FDA authorization for notifying users of breathing patterns suggestive of moderate-to-severe sleep apnea [28]. Commercial wearable devices routinely quantify nocturnal HRV, commonly using RMSSD, and validation studies have demonstrated varying levels of agreement with reference ECG measurements [29,30]. Despite the widespread adoption of consumer wearables for physiological data collection [31], wearable-derived HRV has not yet been established as a screening tool for diabetic microvascular complications, including retinopathy, nephropathy, and neuropathy. This represents an important translational opportunity: physiological signals already collected by consumer wearables could potentially be repurposed for diabetes-complication risk assessment.

NeuroKit2 parameterizes the SDANN time window, introducing variants such as SDANN1 (1-min segments) and SDANN2 (2-min segments) [32]. To date, few studies have examined the discriminative value of SDANN1 and SDANN2 in clinical applications. If minute-scale SDANN indices prove discriminative for DR, they could in principle be computed from the same sleep recordings already captured by consumer wearables, enabling passive, equipment-free DR pre-screening at population scale.

### 1.5. Study Objectives and Hypothesis

This exploratory pilot study investigates, in a small but well-characterized male inpatient cohort, whether sleep-onset minute-scale SDANN indices serve as candidate screening biomarkers for DR in T2DM. Specific aims include: (1) evaluating the discriminative performance of SDANN1 for DR using non-parametric AUROC analysis and determining the optimal screening threshold; (2) quantifying DR risk through odds ratios, risk stratification, and the number needed to refer; (3) comparing minute-scale SDANN indices with traditional HRV indices (SDNN, RMSSD) and clinical variables (age, mean heart rate) to identify the source of discriminative signal; and (4) characterizing the optimal observation window (sleep-onset transition vs. later sleep periods) and minimum recording duration for SDANN-based screening.

## 2. Materials and Methods

### 2.1. Data Source

This study is reported in accordance with the Standards for Reporting Diagnostic Accuracy (STARD) 2015 guideline [33]; a completed checklist is provided in Supplementary Table S1.

**Figure 1:**
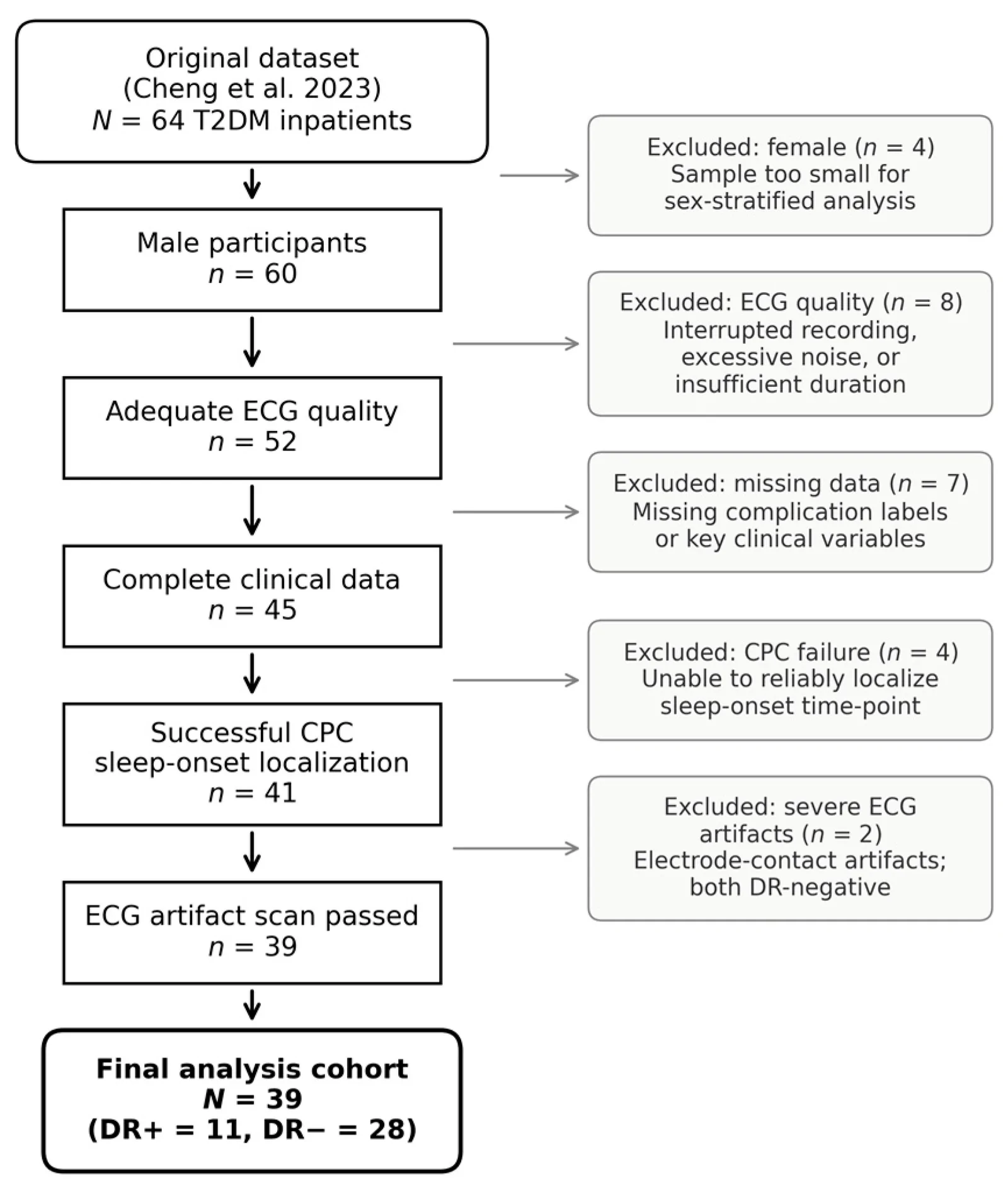
Participant selection flow diagram. Participants were drawn from the publicly available dataset of Cheng et al. [22]. Following sequential eligibility and signal-quality screening, 39 participants were included in the final analysis. Downward arrows indicate the progression of participants through successive screening steps; horizontal arrows indicate participants excluded at each step. CPC, cardiopulmonary coupling; DR, diabetic retinopathy; ECG, electrocardiography.

This study used the dataset published by Cheng W et al. on Mendeley Data (DOI: 10.17632/9c47vwvtss.4, CC BY 4.0) [22]. The original dataset contains 64 T2DM inpatients. The participant selection flow is shown in Figure 1.

ECG was recorded with a Holter recorder by DynaDx Corporation (sampling rate 250 Hz) continuously for 24 h. Sleep-onset time-points were determined by the original dataset authors using cardiopulmonary coupling (CPC) analysis [22,34], with sleep onset localized based on an increase in the proportion of high-frequency coupling (HFC). The present study directly used the CPC analysis results provided by the original dataset.

Clinical data include fasting glucose, glycated hemoglobin (HbA1c), blood pressure, blood lipids, liver and renal function, urinary indicators, and binary labels (yes/no) for six diabetic complications. The original dataset does not provide duration of diabetes, which is an important limitation of this study (see Section 4.4).

Ethics statement: The original dataset was approved by the Institutional Review Board of Suzhou Science & Technology Town Hospital (approval number IRB2019045), with participants recruited between July 2019 and January 2021; written informed consent was obtained from all participants. The present study performs secondary analysis on de-identified, publicly available data. The present analysis was reviewed by the Institutional Review Board of National Yang Ming Chiao Tung University and determined to be exempt from full ethical review (approval code NYCU115232AW; 15 September 2026), as it involves only secondary analysis of a publicly available, fully de-identified dataset.

### 2.2. Study Population

The original dataset includes binary labels for six diabetic complications. DR was selected as the target condition because it had the largest number of positive cases and a direct pathophysiological link to autonomic dysfunction. After applying inclusion and exclusion criteria, the final analyzed sample comprised *N* = 39 (DR-positive 11, DR-negative 28; DR prevalence 28.2%). All 39 participants were male; the original dataset included only four female patients, an insufficient sample for sex-stratified analysis, and they were excluded during participant selection. This sex limitation is discussed in Section 4.4. A formal a priori sample-size calculation was not performed; the sample size was fixed by the available public dataset, consistent with the exploratory, hypothesis-generating design. With 11 events and a single pre-specified primary predictor, the small number of events provides limited precision and would permit reliable detection only of relatively large effects; the wide confidence intervals around the AUROC (0.530–0.901) and odds ratio (1.7–38.4) reflect this limitation (Section 4.4).

### 2.3. HRV Feature Extraction

Using the CPC-derived sleep-onset time-points provided by the original dataset, we extracted 30 min of continuous ECG immediately after sleep onset. NeuroKit2 [32] was used for R-wave detection and HRV feature computation. The following candidate predictors were selected a priori based on physiological rationale:

**Minute-scale indices (hypothesized):** SDANN1, SDANN2, SDANN5—standard deviation of segment-mean RR intervals computed with 1-, 2-, and 5-min non-overlapping segments, respectively.

**Traditional time-domain benchmarks:** SDNN (standard deviation of all RR intervals), RMSSD (root mean square of successive RR differences), SDNN/RMSSD ratio.

**Frequency-domain indices:** LF power (0.04–0.15 Hz), HF power (0.15–0.4 Hz), LF/HF ratio. Power spectral density was estimated using Welch’s periodogram (NeuroKit2 default parameters) and reported as absolute power (ms^2^). Because all between-group comparisons used non-parametric methods (Mann-Whitney U test and non-parametric AUROC), which are invariant to monotonic transformations, natural-log transformation of frequency-domain indices was not applied.

**Basic cardiac parameters:** mean heart rate, mean NN interval.

**Demographic reference: age.**

**Figure 2:**
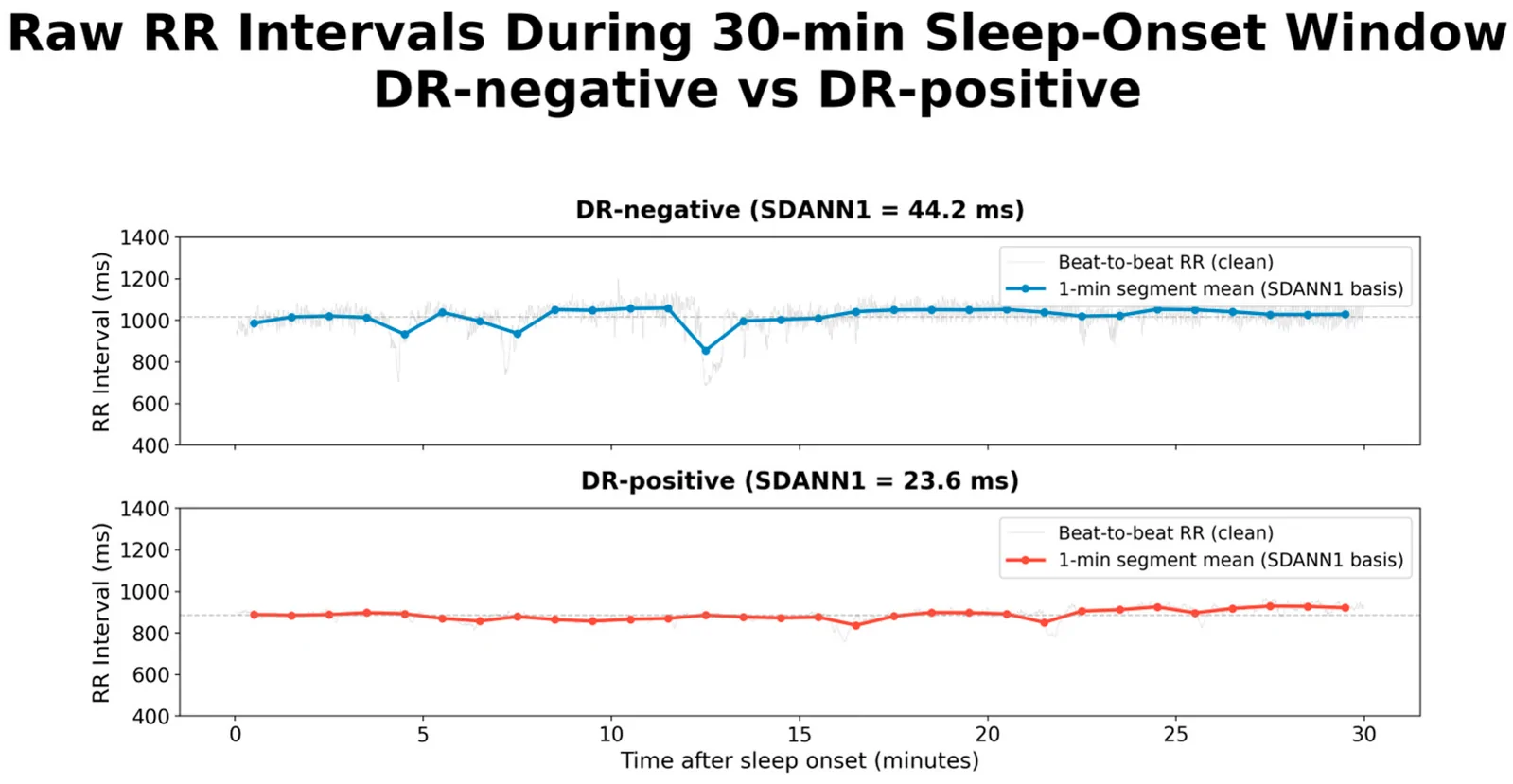
Raw RR intervals during the 30-min sleep-onset window for a DR-negative participant (upper panel, SDANN1 = 44.2 ms; near the DR− group median of 44.8 ms) and a DR-positive participant (lower panel, SDANN1 = 23.6 ms; equal to the DR+ group median). Gray traces: beat-to-beat RR intervals; colored lines: 1-min segment means (the basis for SDANN1 computation). This contrast illustrates the characteristic reduction in minute-scale heart rate variability in the DR+ group quantified by SDANN1. DR, diabetic retinopathy; RR, interval between successive R waves; SDANN1, standard deviation of the averages of normal-to-normal intervals calculated using 1-min segments.

Computation of SDANN: taking SDANN1 as an example, the 30-min RR (beat-to-beat)-interval sequence is divided into 30 non-overlapping 1-min segments. The mean of all RR intervals within each segment is computed, and the standard deviation of these 30 segment means is then computed. SDANN2 and SDANN5 are computed analogously using 2- and 5-min segments. SDANN captures the trend of heart-rate fluctuations on a minute scale rather than instantaneous beat-to-beat variations. Figure 2 illustrates the raw RR intervals and 1-min segment means for a representative DR-negative and DR-positive participant (the latter at the DR+ group median), showing the contrast in minute-scale variability that SDANN1 quantifies. Group-level distributions are shown in Table 1.

Minute-scale SDANN variants were pre-specified on physiological grounds: because averaging RR intervals within each segment suppresses oscillations faster than the segment length—including respiratory-frequency (HF-band) and baroreflex-frequency (LF-band) oscillations—SDANN isolates heart-rate fluctuations on the minute scale and slower, the long-term variance components it was designed to estimate [12]. Minute-scale SDANN therefore targets the slow autonomic dynamics engaged during the sleep-onset transition (Section 1.3 and Section 1.4) [18,20]. SDANN1 was pre-specified as the primary biomarker because 1-min segments yield 30 data points per 30-min recording—five times more than SDANN5 (6 segments)—providing the most stable SD estimate. SDANN2 is reported as a segmentation robustness check (Section 3.5). Throughout this manuscript, SDANN1, SDANN2, and SDANN5 denote 1-, 2-, and 5-min-segment variants computed over the 30-min sleep-onset window; they are explicitly distinguished from the conventional Task Force SDANN, which uses 5-min segments over 24-h recordings [12].

### 2.4. ECG Quality Assessment and Exclusions

A systematic ECG artifact scan was performed on all 41 eligible participants’ recordings. Each recording was evaluated for four artifact types: (a) abnormal RR intervals (<300 ms or >2000 ms), (b) ECG amplitude spikes exceeding 50,000 units (approximately 100× normal range), (c) signal gaps (>5 s between successive R-peaks), and (d) successive RR-interval differences exceeding 50% of the preceding interval. Severity was classified as CLEAN, LOW, MODERATE, or HIGH based on the number and type of artifacts detected.

Two participants (both DR-negative) were classified as HIGH severity with clinically significant electrode-contact artifacts: one showed 52 ECG amplitude spikes (peak amplitude 799,399 units, approximately 600× normal) with 5 signal dropouts, and the other showed 39 amplitude spikes with 1 signal dropout. These two participants were excluded from the analysis, yielding a final sample of *N* = 39.

For the remaining 39 participants, beat-level artifact filtering was applied prior to HRV computation: individual RR intervals were excluded if they met any of the four artifact criteria (a–d) above. Excluded beats were removed from the RR series before computing segment means and SDANN values. The median rejection rate was 0.2% of beats per recording (range 0–6.2%), confirming minimal data loss.

### 2.5. Statistical Analysis

The primary analysis adopted a biomarker discovery and clinical screening framework:

**Biomarker comparison.** SDANN1 was pre-specified as the primary candidate biomarker based on the physiological rationale that minute-scale heart-rate regulation during the sleep-onset transition would be most sensitive to DAN-related autonomic impairment (see Section 1.3 and Section 1.4). Eleven additional predictors (SDANN2, SDANN5, SDNN, RMSSD, SDNN/RMSSD ratio, LF power, HF power, LF/HF ratio, mean heart rate, mean NN interval, and age) were evaluated as secondary comparators to contextualize the SDANN1 signal. Non-parametric area under the receiver operating characteristic curve (AUROC) was computed for each candidate predictor using the raw predictor value as the discriminant score. Significance was assessed by Mann-Whitney U test. The 95% confidence interval (CI) for AUROC was computed by bootstrap resampling (2000 resamples). Because this is an exploratory pilot study with a single pre-specified primary predictor, no multiplicity adjustment was applied to the secondary comparisons; their *p*-values should be interpreted descriptively.

**Group comparison.** Between-group differences (DR+ vs. DR−) were tested by Mann-Whitney U test. Effect sizes were quantified by Cohen’s d and Glass’s Δ. The 95% CI for the mean group difference was estimated by bootstrap (10,000 resamples). Cohen’s d was computed using the pooled standard deviation, and Glass’s Δ using the DR-positive group standard deviation as the reference denominator; both are reported for the primary comparison in Section 3.2.

**Optimal threshold determination.** Youden’s J index (sensitivity + specificity − 1) was used to determine the optimal SDANN1 screening threshold from the ROC curve.

**Screening metrics.** At the optimal threshold: sensitivity, specificity, positive predictive value (PPV), negative predictive value (NPV), positive likelihood ratio (LR+), and negative likelihood ratio (LR−).

**Risk quantification.** Odds ratio (OR) with 95% CI (Woolf’s method), relative risk (RR), and Fisher’s exact test for the 2 × 2 contingency table defined by the optimal threshold. The number of screen-positive individuals requiring confirmatory assessment to identify one DR case was calculated as 1/PPV and is reported as the number needed to refer (NNR); this quantity is distinct from the conventional population-level number needed to screen.

**Risk stratification.** Patients were stratified into three tiers based on SDANN1: high risk (≤25 ms), moderate risk (25–45 ms), and low risk (>45 ms). DR prevalence and the proportion of DR-positive cases captured were computed for each tier.

**Adjusted association.** To complement the univariate analysis, Firth’s penalized logistic regression [35] was used to estimate SDANN1’s adjusted association with DR, controlling sequentially for age, HbA1c, and the apnea–hypopnea index (AHI; available for 36 of 39 participants). All covariates were standardized; the resulting odds ratios reflect the change per 1-SD increase. The primary evidence for SDANN1’s discriminative value rests on the non-parametric AUROC and Fisher’s exact test; multivariable models are presented as supplementary analyses.

**Multi-threshold analysis.** A reference table of screening metrics at thresholds from ≤15 ms to ≤40 ms was computed to enable clinical threshold selection balancing sensitivity and specificity requirements.

**Pairwise AUROC comparison.** DeLong tests [36] were used to compare SDANN1’s AUROC with those of RMSSD and SDNN.

**Internal validation.** Optimism-corrected AUROC was estimated with the standard bootstrap optimism-correction procedure (2000 resamples) [37]: a logistic model refitted in each bootstrap sample was applied to the original sample, and the mean optimism was subtracted from the apparent AUROC; the .632/.632+ estimators were not used. The Monte Carlo standard error of the optimism estimate was approximately 0.002, confirming that 2000 resamples provide adequate precision at *N* = 39. As an additional check on threshold-selection overfitting, leave-one-out cross-validation (LOO-CV) was performed, re-deriving the Youden-optimal threshold within each training fold and applying it to the held-out participant (Section 3.2, Table S3).

### 2.6. Duration-Effect and Time-Window Analyses

To characterize the optimal recording parameters, two supplementary analyses were conducted:

**Duration-effect analysis.** Fixing the starting point at sleep onset, ECG segments of 5, 10, 15, 20, 30, and 60 min were extracted. SDANN1 was computed from each segment and its non-parametric AUROC for DR detection was evaluated.

**Time-window analysis.** Fixing the duration at 30 min, ECG segments from 0–30, 30–60, 60–90, and 90–120 min after sleep onset were extracted for AUROC comparison.

### 2.7. Software

All analyses were performed in Python 3.10.13 with the following packages: NeuroKit2 0.2.12 for ECG processing and HRV feature extraction, firthlogist 0.5.0 for Firth’s penalized logistic regression, scikit-learn 1.7.2 for ROC analysis, NumPy 1.26.4, pandas 2.2.3, and SciPy 1.14.1 for numerical computation and statistical testing, and matplotlib 3.10.8 for figure generation.

## 3. Results

### 3.1. Biomarker Screening: Only SDANN Discriminates DR Status

Non-parametric AUROC analysis across 12 candidate predictors revealed that only the SDANN family reached statistical significance for DR detection (Table 2): SDANN1 (AUROC = 0.731, *p* = 0.028), SDANN5 (0.731, *p* = 0.028), and SDANN2 (0.721, *p* = 0.035). The three SDANN variants showed near-identical performance, with overlapping 95% CIs. Traditional time-domain indices (RMSSD: 0.688, *p* = 0.073; SDNN: 0.672, *p* = 0.101), frequency-domain indices (LF: 0.597, *p* = 0.357; HF: 0.532, *p* = 0.767; LF/HF: 0.584, *p* = 0.426), mean heart rate, mean NN interval, SDNN/RMSSD ratio, and age did not discriminate DR status.

All time-domain HRV indices (SDNN, RMSSD, SDANN1/2/5) were computed from artifact-filtered RR series; frequency-domain indices (LF, HF, LF/HF) and age used raw values. Only the SDANN family reached statistical significance. RMSSD and SDNN approached borderline significance but did not reach *p* < 0.05. AUROC, area under the receiver operating characteristic curve; CI, confidence interval; DR, diabetic retinopathy; HF, high-frequency power; HR, heart rate; HRV, heart rate variability; LF, low-frequency power; MeanNN, mean normal-to-normal interval; RMSSD, root mean square of successive differences; SDANN, standard deviation of the average normal-to-normal intervals; SDNN, standard deviation of normal-to-normal intervals.SDANN1, SDANN calculated using 1-min segments; SDANN2, SDANN calculated using 2-min segments; SDANN5, SDANN calculated using 5-min segments.

DeLong tests comparing SDANN1 with RMSSD (Δ AUROC = 0.042, z = 0.45, *p* = 0.655) and SDNN (Δ AUROC = 0.058, z = 1.14, *p* = 0.257) were non-significant, as expected given the limited statistical power at *N* = 39 to resolve AUROC differences of this magnitude (Table S2). The convergent pattern—three SDANN variants all significant, all nine non-SDANN predictors non-significant—provides stronger evidence than any single pairwise comparison. Under a conservative Bonferroni correction treating all 12 candidates as co-primary (α = 0.05/12 ≈ 0.004), no individual predictor—including SDANN1 (*p* = 0.028)—would remain statistically significant. Because SDANN1 was pre-specified as the primary biomarker and the remaining 11 predictors served as descriptive comparators (Section 2.5), no multiplicity adjustment was applied; accordingly, these results should be interpreted as hypothesis-generating evidence rather than confirmatory findings.

The three SDANN variants are highly collinear (Spearman ρ = 0.945–0.985) and their convergent significance serves as a segmentation consistency check: the signal is not an artifact of a specific segment length but is robust across parameterizations of the same underlying minute-scale autonomic phenomenon.

### 3.2. SDANN1 as the Primary Biomarker: Group Comparison and Discriminative Performance

**Figure 3:**
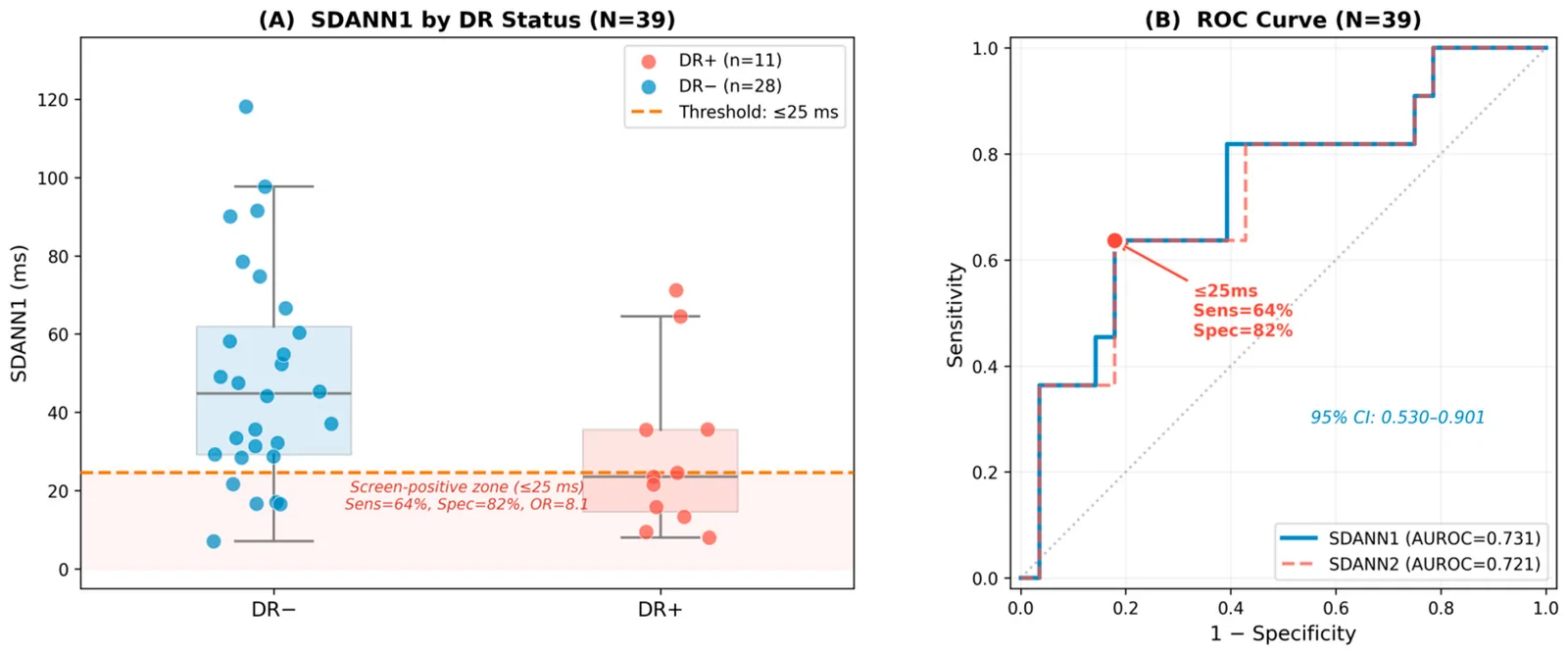
(**A**) SDANN1 distribution by DR status (*N* = 39). Each dot represents one participant. Box plots show median and interquartile range. The dashed line indicates the optimal screening threshold (≤25 ms). The shaded area below the threshold marks the screen-positive zone. (**B**) Receiver operating characteristic (ROC) curves for SDANN1 (AUROC = 0.731) and SDANN2 (AUROC = 0.721). The red dot marks the Youden-optimal operating point for SDANN1 (sensitivity 63.6%, specificity 82.1%). AUROC, area under the receiver operating characteristic curve; DR+, diabetic retinopathy present; DR−, diabetic retinopathy absent; SDANN1, standard deviation of the averages of normal-to-normal intervals calculated using 1-min segments; SDANN2, standard deviation of the averages of normal-to-normal intervals calculated using 2-min segments.

SDANN1 was significantly lower in DR-positive than DR-negative patients (Table 1, Figure 3A): mean 29.4 ± 21.1 vs. 48.7 ± 27.7 ms; median 23.6 [interquartile range (IQR): 14.6–35.6] vs. 44.8 [29.2–61.9] ms (Mann-Whitney U = 83.0, *p* = 0.028). Cohen’s d = 0.74 (medium-to-large effect); Glass’s Δ = 0.91. The bootstrap 95% CI for the group mean difference was 2.8–34.6 ms.

SDANN1 yielded a non-parametric AUROC of 0.731 (95% CI: 0.530–0.901) for DR detection (Figure 3B); bootstrap-optimism correction confirmed minimal overfitting (corrected AUROC = 0.727; Monte Carlo standard error ≈ 0.002; Table S3). Youden’s J index identified ≤ 25 ms as the optimal screening threshold (J = 0.458), with screening performance shown in Table 3a. A multi-threshold reference table is provided in Table 3b for clinical flexibility. To further address threshold-selection overfitting, leave-one-out cross-validation—re-deriving the Youden-optimal threshold within each training fold—yielded a sensitivity of 54.5% (6/11) and specificity of 82.1% (23/28); the fold-specific thresholds were highly stable (range 23.6–24.6 ms; median 24.6 ms), and the LOO-CV AUROC of the corresponding logistic model was 0.653 (Table S3). Threshold-level metrics in Table 3a,b are apparent values and should be interpreted accordingly.

Threshold-specific metrics are apparent values from the same dataset used for threshold selection, without optimism correction; actual performance may be lower. DR, diabetic retinopathy; LR+, positive likelihood ratio; LR−, negative likelihood ratio; NPV, negative predictive value; PPV, positive predictive value; SDANN1, standard deviation of the averages of normal-to-normal intervals calculated using 1-min segments.

The ROC-optimal threshold (≤25 ms) maximizes the Youden’s J index of the ROC curve. LR+, positive likelihood ratio; LR−, negative likelihood ratio; NPV, negative predictive value; PPV, positive predictive value; ROC, receiver operating characteristic; SDANN1, standard deviation of the averages of normal-to-normal intervals calculated using 1-min segments.

### 3.3. Risk Quantification: Odds Ratio and Clinical Significance

At the optimal threshold of ≤25 ms, the DR prevalence among screen-positive patients was 58.3% (7/12), compared with 14.8% (4/27) among screen-negative patients (Table 4a). Results are as follows:

Patients with SDANN1 ≤ 25 ms had 8.1-fold higher odds of DR compared with those above the threshold. The NNR of 2.3 indicates that among patients flagged as screen-positive, approximately every second to third patient would have DR—an efficient yield for a pre-screening triage tool. Because PPV and NNR are prevalence-dependent, these values reflect the sample DR prevalence of 28.2%. The positive likelihood ratio (LR+ = 3.56) is prevalence-independent and can be applied via Bayes’ theorem. Based on the global prevalence estimate of 34.6% among individuals with diabetes [1], the expected PPV would be approximately 66%; at 20%, approximately 47%. In all scenarios, SDANN1 screening would substantially enrich the referred population relative to unselected screening.

### 3.4. Risk Stratification: Three-Tier Clinical Framework

**Figure 4:**
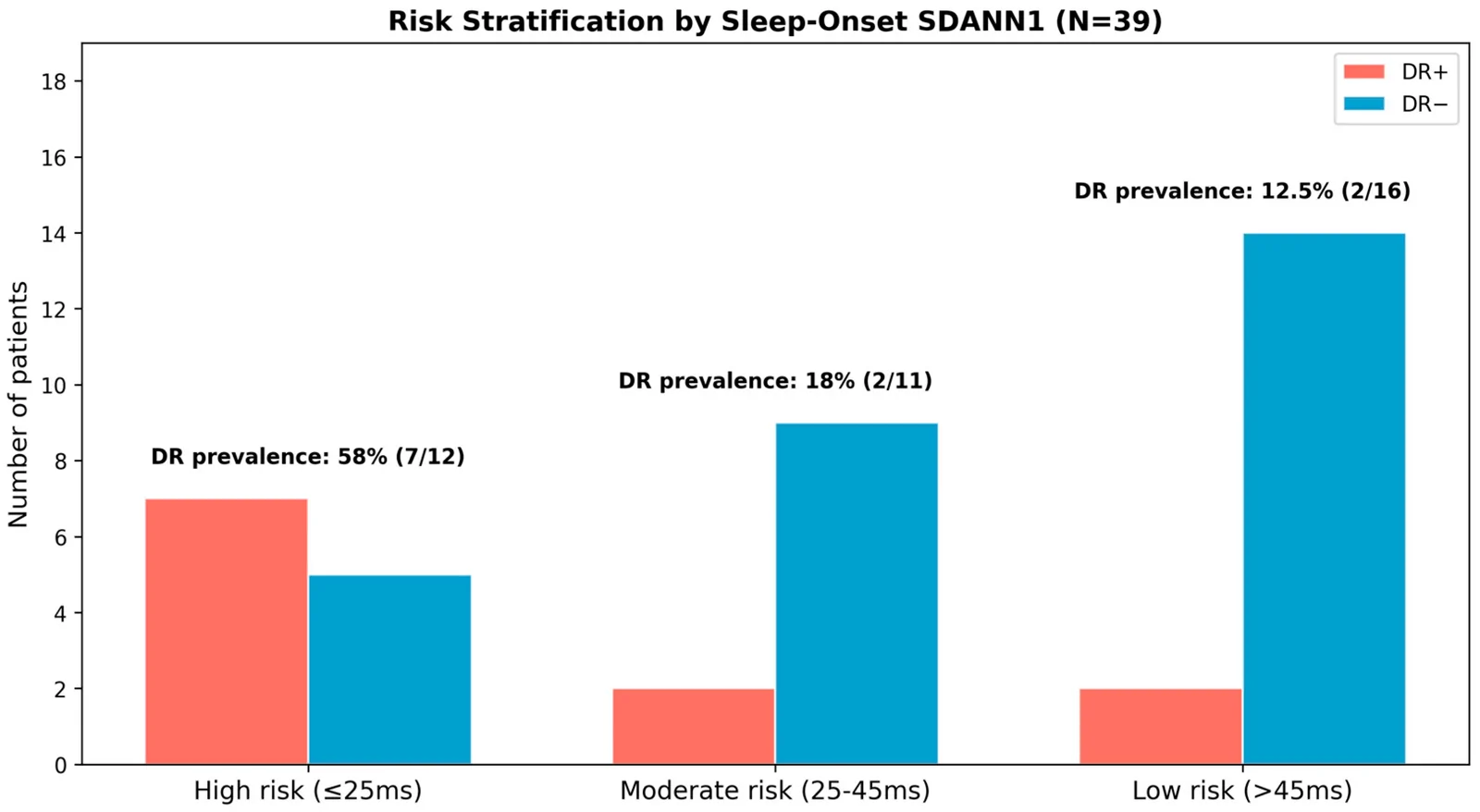
Risk stratification by sleep-onset SDANN1 (*N* = 39). Patients were classified as high risk (≤25 ms), moderate risk (25–45 ms), and low risk (>45 ms). Bar heights indicate the number of DR-positive (red) and DR-negative (blue) patients per tier. DR prevalence annotations show the gradient from 58% (high risk) to 12.5% (low risk). DR, diabetic retinopathy; SDANN1, standard deviation of the averages of normal-to-normal intervals calculated using 1-min segments.

To evaluate clinical utility beyond a single threshold, patients were stratified into three risk tiers based on SDANN1 (Table 4b, Figure 4). The high-risk boundary (≤25 ms) corresponds to the Youden-optimal threshold; the low-risk boundary (>45 ms) was set at the DR-negative group median (44.8 ms, rounded to 45 ms), above which DR prevalence approaches background levels:

The high-risk tier captured 63.6% (7/11) of all DR-positive patients with a DR prevalence of 58.3%—more than double the overall sample prevalence (28.2%).

The high-risk tier (SDANN1 ≤ 25 ms) captured 63.6% of DR-positive patients, with a DR prevalence of 58.3%—nearly double the sample prevalence of 28.2%. DR, diabetic retinopathy; DR+, diabetic retinopathy present; DR−, diabetic retinopathy absent; SDANN1, standard deviation of the averages of normal-to-normal intervals calculated using 1-min segments.

### 3.5. SDANN2 Segmentation Robustness Check

Because SDANN1 and SDANN2 differ only in segment length (1- vs. 2-min windows applied to the same 30-min RR-interval sequence), they are inherently collinear (Spearman ρ = 0.985) and SDANN2 cannot serve as an independent confirmatory biomarker. Instead, SDANN2 provides a segmentation robustness check: if the signal were an artifact of a specific segment length, SDANN2 would not replicate the finding. SDANN2 yielded AUROC = 0.721 (95% CI: 0.518–0.894), *p* = 0.035, with identical screening performance at its optimal threshold (sensitivity 63.6%, specificity 82.1%; Supplementary Figure S1), confirming that the discriminative signal is robust across segment-length parameterizations.

### 3.6. Adjusted Association

Firth’s penalized logistic regression confirmed SDANN1’s inverse association with DR after covariate adjustment (Table S4). SDANN1 was significantly associated with DR univariably (OR per 1-SD = 0.42, 95% CI: 0.14–0.96, *p* = 0.039), and the association remained stable after adjustment for age (OR = 0.38, 95% CI: 0.11–0.94, *p* = 0.034). Adding HbA1c (OR = 0.45, *p* = 0.086) reduced the available sample to *N* = 27 due to missing values, attenuating statistical power. The consistent point estimate across nested models (OR ≈ 0.38–0.45) supports SDANN1’s independent association with DR. To address obstructive sleep apnea as a potential confounder, AHI was additionally entered as a covariate (*N* = 36 with available AHI). SDANN1’s association remained directionally stable (OR per 1-SD = 0.42, 95% CI: 0.11–1.02, *p* = 0.056), whereas AHI was not statistically associated with DR in these exploratory models (OR = 1.19, 95% CI: 0.60–2.92, *p* = 0.628); in the full model with age, HbA1c, and AHI (*N* = 24), the SDANN1 point estimate was similar (OR = 0.48, 95% CI: 0.12–1.17, *p* = 0.114; Table S4). The wider confidence intervals are consistent with the reduced sample size, and these exploratory models provided no clear evidence that adjustment for AHI materially altered the SDANN1 point estimate. Given the low events-per-variable ratios (2.2–5.5), all multivariable estimates should be regarded as exploratory and illustrative.

### 3.7. Effect of Recording Duration

SDANN1’s discriminative performance improved monotonically with recording duration from 5 to 30 min (Table 5): AUROC rose from 0.519 (5 min, *p* = 0.864) to 0.731 (30 min, *p* = 0.028), reaching borderline significance at 20 min (AUROC = 0.701, *p* = 0.055) and full significance at 30 min. At 60 min, AUROC declined slightly to 0.685 (*p* = 0.078), likely because inclusion of stable-sleep ECG beyond the discriminative sleep-onset window dilutes the signal. SDANN1 computed from only 5 min of ECG (5 segment means) yields an inherently unstable estimate. These results indicate that 30 min of sleep-onset ECG is the minimum for reliable SDANN1-based screening. Because the duration analysis is partly data-driven, the apparent optimum at 30 min could in part reflect small-sample variability; however, the monotonic improvement from 5 to 30 min and the physiologically expected decline once stable-sleep ECG is included argue against a purely spurious peak. This recording parameter will be re-examined in the ongoing external validation study.

SDANN1 AUROC increased monotonically from 5 min (0.519) to 30 min (0.731), reaching statistical significance at 30 min. The decline at 60 min (0.685) reflects inclusion of stable-sleep ECG beyond the discriminative sleep-onset window. SDANN1 computed from only 5 min uses only 5 segment means, yielding an unstable estimate. The 30-min CI is from the primary analysis (10,000 bootstrap resamples); other durations used 2000 resamples. AUROC, area under the receiver operating characteristic curve; CI, confidence interval; DR, diabetic retinopathy; ECG, electrocardiography; SDANN1, standard deviation of the averages of normal-to-normal intervals calculated using 1-min segments.

### 3.8. Effect of Sleep Time Window

The sleep-onset transition (0–30 min) was the only time window where SDANN1 significantly discriminated DR status (AUROC = 0.731, *p* = 0.028; Table 6). AUROC declined progressively through subsequent windows: 0.672 (30–60 min, *p* = 0.101), 0.539 (60–90 min, *p* = 0.720), and 0.594 (90–120 min, *p* = 0.374). By 60–120 min after sleep onset—well beyond the sleep-onset transition—SDANN1 approached chance-level performance. This pattern confirms that the sympatho-vagal transition at sleep onset is the critical observation window: the dynamic regulatory challenge of falling asleep amplifies between-group differences in autonomic capacity, while between-individual differences narrow in later sleep periods.

The sleep-onset transition (0–30 min) was the only window where SDANN1 significantly discriminated DR. AUROC declined progressively in subsequent windows, approaching chance level in the later windows (60–120 min, 0.539–0.594). This confirms the sympatho-vagal transition at sleep onset as the critical observation window. Time windows are defined solely by elapsed time after CPC-derived sleep onset; given substantial inter-individual variability in sleep architecture, no correspondence with specific sleep stages is implied, and the relationship between sleep stages and these results remains uncertain. AUROC, area under the receiver operating characteristic curve; CI, confidence interval; DR, diabetic retinopathy; SDANN1, standard deviation of the averages of normal-to-normal intervals calculated using 1-min segments.

## 4. Discussion

### 4.1. Main Findings

This study has three main findings. First, sleep-onset SDANN1 was the only candidate predictor to discriminate DR status with statistical significance (AUROC = 0.731, *p* = 0.028), while all traditional HRV indices (SDNN, RMSSD), mean heart rate, and age failed to discriminate. This indicates that DR-related autonomic impairment manifests specifically in minute-scale heart-rate regulation during the sleep-onset transition. Second, patients with SDANN1 ≤ 25 ms had higher odds of DR (OR = 8.1, 95% CI: 1.7–38.4; Fisher’s *p* = 0.017), and risk stratification showed a DR prevalence of 58% in the high-risk tier (≤25 ms) versus 12.5% in the low-risk tier (>45 ms)—a 4.7-fold gradient. Third, SDANN1’s discriminative power was concentrated in the sleep-onset window (AUROC 0.731 at 0–30 min vs. 0.539–0.672 in later windows) and required 30 min of recording to reach significance (AUROC 0.701, *p* = 0.055 at 20 min; 0.731, *p* = 0.028 at 30 min), confirming that the physiological challenge of the sympatho-vagal transition is what drives the signal.

Firth’s penalized regression further confirmed SDANN1’s independent inverse association with DR (OR per 1-SD = 0.38–0.45 across nested adjustment models), supporting that the association is not attributable to age or glycemic control (HbA1c) alone.

### 4.2. Why Does the Sleep-Onset Transition Discriminate Better than Stable Sleep?

The sleep-onset transition is the period during which the autonomic nervous system shifts from sympathetic-dominant wakefulness to parasympathetic-dominant sleep [18,19,20]. During this transition, heart rate fluctuates dynamically on the minute scale, and SDANN captures the magnitude of these fluctuations. Once stable sleep is established, heart rate is steadily controlled by parasympathetic activity, between-individual differences narrow, and SDANN’s discriminative power declines.

SDANN at sleep onset can be viewed as an index reflecting the dynamic regulatory capacity of the autonomic nervous system. The time-window analysis in this study supports this view: the sleep-onset transition (0–30 min) demonstrated the best discriminative performance, with progressive decline in subsequent stable-sleep windows.

### 4.3. Why Were Classical HRV Indices Not Discriminative?

RMSSD measures beat-to-beat instantaneous variation, and SDNN measures overall variability over the entire recording—these indices reflect variability within a given time segment. In contrast, SDANN tracks the trend of minute-level heart-rate means, capturing dynamic regulation on the minute scale. The AUROC comparison (Table 2) quantifies this contrast: although RMSSD (0.688, *p* = 0.073) and SDNN (0.672, *p* = 0.101) approached borderline significance, only the SDANN family crossed the significance threshold, and the SDNN/RMSSD ratio (0.529) showed no discriminative power for DR. DeLong tests confirmed that the SDANN1–RMSSD AUROC difference was not statistically significant at *N* = 39 (*p* = 0.655; Table S2); however, non-significance does not imply equivalence—it reflects insufficient power to resolve AUROC differences of this magnitude at *N* = 39.

The time-window analysis (Table 6) provides the most direct evidence that SDANN1 captures a minute-scale physiological phenomenon specific to the sleep-onset transition, rather than reflecting a general HRV reduction. SDANN1’s discriminative power declined progressively from AUROC 0.731 at sleep onset (0–30 min) to 0.539 at 60–90 min—approaching chance-level performance. This window-specificity cannot be explained by overall HRV magnitude: if DR patients simply had globally lower HRV, RMSSD and SDNN would also discriminate, and SDANN1 would discriminate equally well during stable sleep. Instead, the signal is concentrated at the sympatho-vagal transition, consistent with the hypothesized autonomic challenge model (Section 4.2). The convergent pattern—three SDANN variants all significant across three segment parameterizations, all nine non-SDANN predictors non-significant—further supports a qualitative distinction at the index level rather than a quantitative difference in effect size. An alternative frequency-domain interpretation also merits consideration. Because SDANN evaluates the variability of segment-averaged RR intervals, it preferentially reflects fluctuations slower than the conventional LF band, overlapping the very-low-frequency (VLF) range [12]. In a post-hoc analysis, however, VLF power did not discriminate DR status (AUROC = 0.604, *p* = 0.326), and SDANN1 was moderately inversely correlated with VLF power (Spearman ρ = −0.51, *p* = 0.001)—the opposite direction from what would be expected if SDANN1 merely proxied slow-band spectral power. These observations suggest that SDANN1’s discriminative signal is not reducible to greater slow oscillatory power; rather, SDANN1 appears to capture the non-stationary minute-scale trend of heart rate during the descent into sleep—dynamics that stationarity-based spectral estimates do not represent. We agree with the view that DR-related information resides in slow heart-rate dynamics, and the relationship between minute-scale time-domain indices and slow HRV components, including VLF, warrants dedicated study in larger cohorts.

### 4.4. Clinical Positioning and Study Limitations

**Clinical positioning.** SDANN1 can serve as a pre-screening triage tool: in resource-limited settings, a single overnight ECG recording—obtainable via consumer-grade wearable devices—could identify a subset of T2DM patients at elevated risk (SDANN1 ≤ 25 ms) for prioritized ophthalmologic referral, while the remainder could be scheduled for routine screening intervals. In this pilot sample, the NNR of 2.3 suggests that for every two to three patients flagged, one would have DR, supporting clinical feasibility. For context, autonomous AI systems analyzing fundus photographs achieve AUCs of 0.94–0.98 for referable DR [5,6], and multi-disease retinal-imaging frameworks now demonstrate robust external validation at scale [38]. An HRV-based approach cannot, and need not, compete with retinal imaging on discriminative accuracy; its intended role is complementary triage—identifying, from passively collected wearable data and without any imaging equipment, the subset of patients who most urgently need the imaging pathway—rather than an alternative to imaging-based screening. For such a triage role, the relevant benchmark is not the AUC of diagnostic imaging but whether risk stratification meaningfully enriches the referred population: the observed LR+ of 3.56 and the 4.7-fold prevalence gradient across risk tiers provide preliminary evidence of risk enrichment that warrants prospective external validation, while an AUROC in the 0.70–0.75 range remains modest and precludes any immediate deployment.

**Obstructive sleep apnea (OSA).** The sample had a moderate-to-high OSA burden (mean apnea–hypopnea index [AHI] = 25.3 ± 19.6 events/hour, N = 36). AHI-stratified analysis showed comparable SDANN1 discriminative performance across mild and moderate-to-severe OSA subgroups (AUROC 0.736 vs. 0.754; Table S5), and the Spearman correlation between SDANN1 and AHI was weak (ρ = −0.28, *p* = 0.09). These results indicate that SDANN1’s discriminative power for DR is robust across OSA severity strata, rather than driven by a specific subgroup. Consistently, when AHI was entered as a covariate in Firth models (Section 3.6, Table S4), SDANN1’s association remained directionally stable while AHI showed no independent association with DR. Nevertheless, OSA could act as both a confounder and a mediator on the pathway linking autonomic dysfunction to microvascular disease, and these roles cannot be fully disentangled at this sample size.

**Feasibility of wearable deployment.** A 24-h Holter recording is impractical for population screening; however, the proposed measurement requires only 30 min of interbeat-interval data following sleep onset. Consumer wearables already detect sleep onset using accelerometry combined with heart-rate dynamics, and photoplethysmography-derived interbeat intervals during sleep show close agreement with ECG-derived RR intervals, as motion artifact is minimal during sleep [29,30]. SDANN’s two-stage segment-averaging architecture further confers robustness to sporadic beat-detection errors (Section 1.4). Deployment would therefore require only firmware-level extraction of 1-min interbeat-interval means for the first 30 min after device-detected sleep onset—no additional sensors or imaging hardware. Prospective validation of wearable-derived SDANN1 against simultaneous ECG remains necessary before clinical use.

**Study limitations.**

**Small sample and class imbalance:** The sample size (*N* = 39, 11 events) was limited but may be sufficient for exploratory predictor development, as previous methodological work has shown that some prediction problems do not require large training sets for classifier development [39]. The wide confidence intervals around AUROC and OR reflect this limitation. The optimal threshold of ≤ 25 ms and all derived screening metrics were determined and evaluated within the same dataset; an independent external validation study is currently under analysis. Bootstrap optimism correction and LOO-CV (Table S3) constitute internal validation only; external validation in independent cohorts remains the critical next step before any clinical application, in line with recommendations for translational biomarker development [40].

**Diabetes duration uncontrolled:** The original dataset does not provide duration of diabetes. We regard this as a major confounding limitation. Duration of diabetes is a major risk factor for DR; UKPDS 50 reported relative risks of approximately 4.1–8.1 for retinopathy progression across higher HbA1c categories compared with the lowest HbA1c category [41]. To estimate the potential confounding magnitude: if DR-positive patients in this sample had, on average, 5 years longer diabetes duration than DR-negative patients, the duration-attributable OR would be approximately 1.08^5 ≈ 1.5. Even under this assumption, the residual SDANN1 OR after accounting for duration would remain approximately 8.1/1.5 ≈ 5.4, suggesting that a duration imbalance of this magnitude alone would be unlikely to fully explain the observed association. Two additional observations support this assessment. First, the DR-positive and DR-negative groups had nearly identical ages (50.3 vs. 49.2 years, *p* = 0.956; Table 1), suggesting that large between-group differences in diabetes duration—while possible—are not the most parsimonious explanation for the observed SDANN1 difference. Second, Firth-adjusted ORs remained stable after age adjustment (OR 0.38–0.45; Table S4). Our ongoing external validation study includes duration of diabetes as a covariate and will directly address this limitation. HbA1c partially proxies cumulative glycemic exposure but reflects only recent (8–12 week) glycemia, and medication data were not available in the dataset; notably, HbA1c did not differ significantly between groups (9.0 vs. 8.3%, *p* = 0.281; Table 1) and its inclusion did not materially alter SDANN1’s odds ratio (Table S4).

**Limited multivariable adjustment:** Although Firth’s penalized regression provides preliminary adjusted ORs (Section 3.6 and Table S4), the EPV values of approximately 2.2–5.5 across the multivariable models are well below conventional recommendations (≥10), and these estimates should be regarded as illustrative. The small number of events also precludes inclusion of additional plausible confounders such as duration of diabetes (not available), blood pressure, lipid profile, and use of antihypertensive or hypoglycemic medication. Future studies in larger samples should report multivariable-adjusted odds ratios with these additional covariates and adequate EPV.

**Cross-sectional design:** This study establishes cross-sectional associations between SDANN and DR and therefore supports SDANN1 only as a candidate risk-stratification biomarker, not as a validated screening test; demonstrating temporal predictive value requires prospective longitudinal validation.

**Non-independent sleep-onset determination and possible sleep-architecture differences:** Sleep onset was determined by CPC analysis, which is itself derived from heart-rate variability and ECG-derived respiration; the definition of the observation window is therefore not fully independent of the HRV signal subsequently analyzed. CPC-based staging shows good agreement with polysomnography [34], and the feature used for onset localization (high-frequency coupling) reflects cardiopulmonary coherence rather than the minute-scale variance quantified by SDANN; nevertheless, polysomnography-based determination of sleep onset would provide stronger, fully independent validation. Relatedly, diabetes and diabetic autonomic neuropathy may themselves alter sleep architecture and the timing of sleep onset, so CPC-determined onset and subsequent sleep structure could differ systematically by DR status, acting as a confounder or a mediator of the observed association; growing evidence links sleep physiology to ocular health [42]. Polysomnography-based studies are needed to disentangle these possibilities.

**Limited generalizability:** Male-only, inpatient, single-center, single-ethnicity (mainland China) sample; results cannot be directly extrapolated to women (who account for nearly half of T2DM patients globally [43]) or to community-based screening settings. HRV exhibits well-documented sex differences—women show relatively higher vagally mediated HRV and different age-related trajectories [44]—so the discriminative threshold, and possibly the effect size, of SDANN1 may differ in women. Inpatient status may further limit generalizability, as acute illness, medication changes, and the hospital sleep environment can all alter HRV and sleep architecture. Our ongoing validation study uses a sex-balanced, community-based design.

## 5. Conclusions

This exploratory pilot study identifies sleep-onset SDANN1 as a candidate risk-stratification biomarker for diabetic retinopathy in T2DM. Among 39 male inpatients, SDANN1 yielded an AUROC of 0.731 (95% CI: 0.530–0.901), with an odds ratio of 8.1 (95% CI: 1.7–38.4; Fisher’s *p* = 0.017) at the optimal screening threshold. Risk stratification showed a DR prevalence gradient from 58% (SDANN1 ≤ 25 ms) to 12.5% (>45 ms), supporting SDANN1’s potential as a pre-screening triage tool, pending external and prospective longitudinal validation. Among 12 candidate predictors, only the SDANN family—reflecting minute-scale autonomic regulation during the sleep-onset transition—discriminated DR status; traditional HRV indices, mean heart rate, and age did not.

An independent external validation study using a larger, multi-site cohort—including duration of diabetes, sex-balanced design, and community-based recruitment—is under analysis and will be reported separately.

## Figures and Tables

**Table 1:** Participant baseline characteristics and subgroup comparisons (*N* = 39).

Characteristic	All (*N* = 39)	DR-Positive (*n* = 11)	DR-Negative (*n* = 28)	*p Value*
Age (years)	49.5 ± 15.8	50.3 ± 17.6	49.2 ± 15.3	0.956
BMI (kg/m^2^)	26.7 ± 4.1	26.1 ± 4.8	26.9 ± 3.8	0.674
HbA1c (%)	8.5 ± 1.7	9.0 ± 1.7	8.3 ± 1.7	0.281
Fasting glucose (mmol/L)	9.2 ± 3.1	10.7 ± 3.4	8.6 ± 2.8	0.124
Systolic BP (mmHg)	134.8 ± 14.3	138.1 ± 16.1	133.5 ± 13.5	0.446
AHI (events/hour) †	25.3 ± 19.6	30.9 ± 27.9	22.8 ± 14.7	0.387
SDANN1 (ms)	43.3 ± 27.2	29.4 ± 21.1	48.7 ± 27.7	0.028 *
SDANN2 (ms)	40.6 ± 27.3	26.8 ± 21.7	46.1 ± 27.7	0.035 *

* *p* < 0.05, Mann-Whitney U test. † AHI available for 36 of 39 participants. DR, diabetic retinopathy; BMI, body mass index; BP, blood pressure; AHI, apnea–hypopnea index. Duration of diabetes is not available in the original dataset (see Section 4.4).

**Table 2:** Single-predictor AUROC comparison for DR detection (*N* = 39).

Rank	Predictor	AUROC	95% Bootstrap CI	Mann-Whitney *p*	Significant
1	SDANN1	0.731	0.530–0.901	0.028	Yes
1	SDANN5	0.731	0.515–0.911	0.028	Yes
3	SDANN2	0.721	0.518–0.894	0.035	Yes
4	RMSSD	0.688	0.494–0.847	0.073	No
5	SDNN	0.672	0.476–0.847	0.101	No
6	Mean HR	0.640	0.451–0.823	0.185	No
6	MeanNN	0.640	0.458–0.827	0.185	No
8	LF power	0.597	0.384–0.789	0.357	No
9	LF/HF ratio	0.584	0.370–0.782	0.426	No
10	HF power	0.532	0.328–0.729	0.767	No
10	SDNN/RMSSD	0.529	0.331–0.722	0.791	No
12	Age	0.502	0.293–0.722	1.000	No

**Table 3:** (**a**). SDANN1 screening performance at the optimal threshold (≤25 ms). (**b**). SDANN1 multi-threshold screening performance.

(**a**)							
**Metric**				**Value**			
Sensitivity				63.6% (7/11)			
Specificity				82.1% (23/28)			
PPV				58.3% (7/12)			
NPV				85.2% (23/27)			
LR+				3.56			
LR−				0.44			
Youden’s J				0.458			
(**b**)							
**Threshold**	**Sensitivity**	**Specificity**	**PPV**	**NPV**	**LR+**	**LR−**	**Youden’s J**
≤15 ms	27.3%	96.4%	75.0%	77.1%	7.64	0.75	0.237
≤20 ms	36.4%	85.7%	50.0%	77.4%	2.55	0.74	0.221
≤23 ms	45.5%	82.1%	50.0%	79.3%	2.55	0.66	0.276
≤25 ms (ROC optimal)	63.6%	82.1%	58.3%	85.2%	3.56	0.44	0.458
≤30 ms	63.6%	71.4%	46.7%	83.3%	2.23	0.51	0.351
≤35 ms	63.6%	60.7%	38.9%	81.0%	1.62	0.60	0.244
≤40 ms	81.8%	53.6%	40.9%	88.2%	1.76	0.34	0.354

**Table 4:** (**a**). Distribution of DR status and association measures at an SDANN1 threshold of ≤25 ms. (**b**). SDANN1 risk stratification.

(**a**)						
	**SDANN1 ≤ 25 ms**		**SDANN1 > 25 ms**		**Total**	
**Contingency table**						
DR+	7		4		11	
DR−	5		23		28	
Total	12		27		39	
**Association measures**						
**Measure**	**Estimate**		**95% CI**		***p*** **-value**	
Odds ratio	8.1		1.7–38.4		0.017 ^1^	
Relative risk	3.9		*—*		*—*	
NNR	2.3		*—*		*—*	
(**b**)						
**Risk Tier**	**SDANN1 Range**	***N***	**DR+**	**DR−**	**DR Prevalence**	**DR+ Captured**
High risk	≤25 ms	12	7	5	58.3% (7/12)	63.6% (7/11)
Moderate risk	25–45 ms	11	2	9	18.2% (2/11)	18.2% (2/11)
Low risk	>45 ms	16	2	14	12.5% (2/16)	18.2% (2/11)

^1^ Fisher’s exact test. CI, confidence interval; DR+, diabetic retinopathy present; DR−, diabetic retinopathy absent; NNR, number needed to refer (the number of screen-positive individuals requiring confirmatory assessment to identify one DR case); SDANN1, standard deviation of the mean normal-to-normal intervals across 1-min segments.

**Table 5:** Recording duration and SDANN1 discriminative performance (*N* = 39).

Duration	AUROC	95% Bootstrap CI	Mann-Whitney *p*	Significant
5 min	0.519	0.284–0.750	0.864	No
10 min	0.568	0.373–0.750	0.522	No
15 min	0.636	0.439–0.817	0.195	No
20 min	0.701	0.500–0.880	0.055	Borderline
30 min	0.731	0.530–0.901	0.028	Yes
60 min	0.685	0.473–0.869	0.078	Borderline

**Table 6:** Sleep time window and SDANN1 discriminative performance (*N* = 39).

Window	AUROC	95% Bootstrap CI	Mann-Whitney *p*	Significant
0–30 min	0.731	0.530–0.901	0.028	Yes
30–60 min	0.672	0.463–0.848	0.101	No
60–90 min	0.539	0.324–0.752	0.720	No
90–120 min	0.594	0.360–0.812	0.374	No

## Data Availability

The dataset used in this study is publicly available on Mendeley Data (DOI: 10.17632/9c47vwvtss.4, CC BY 4.0).

## Supplementary Materials

The following supporting information can be downloaded at: https://www.mdpi.com/article/10.3390/diagnostics16203301/s1, Table S1: STARD 2015 checklist; Table S2: DeLong tests comparing SDANN1 with traditional HRV indices; Table S3: Internal validation—bootstrap-optimism-corrected AUROC; Table S4: Firth penalized logistic regression; Table S5: AHI-stratified analysis; Figure S1: SDANN2 distribution and ROC curve.
