# Supplementary material for: Minute-Scale Sleep-Onset HRV as a Candidate Risk-Stratification Biomarker for Diabetic Retinopathy: An Exploratory Pilot Study
Source: Diagnostics (Basel). 2026 Oct 11;16(20):3301. doi: 10.3390/diagnostics16203301 (PMC13703224; doi:10.3390/diagnostics16203301)
Supplement: Supplementary file 1 [file diagnostics-16-03301-s001.zip › diagnostics-4557406-supplementary.pdf]

diagnostics

Supplementary Materials

MDPI

Minute-Scale Sleep-Onset HRV as a Candidate Screening Biomarker for Diabetic Retinopathy: An Exploratory Pilot Study

Yi-Ting Cheng[1], Yu-Cheng Lin[1], Yu-Ting Ho[1], Terry B.J. Kuo[1,2,4,5*], Cheryl C.H. Yang[1,2,3,5*]

[1] Institute of Brain Science, National Yang Ming Chiao Tung University, Taipei, Taiwan.
[2] Sleep Research Center, National Yang Ming Chiao Tung University, Taipei, Taiwan.
[3] Brain Research Center, National Yang Ming Chiao Tung University, Taipei, Taiwan.
[4] Tsaotun Psychiatric Center, Ministry of Health and Welfare, Nantou, Taiwan.
[5] Department of Education and Research, Taipei City Hospital, Taipei, Taiwan.
* Corresponding authors:

Cheryl C.H. Yang, Institute of Brain Science, National Yang Ming Chiao Tung University, No. 155, Sec. 2, Li-Nong St., Beitou, Taipei 11221, Taiwan. Tel: +886-2-2826-7058; E-mail: cchyang@nycu.edu.tw
Terry B.J. Kuo, Institute of Brain Science, National Yang Ming Chiao Tung University, No. 155, Sec. 2, Li-Nong St., Beitou, Taipei 11221, Taiwan. Tel: +886-2-2826-7000 ext. 66155; E-mail: tbjkuo@nycu.edu.tw

Keywords: heart rate variability; minute-scale HRV; SDANN; diabetic retinopathy; autonomic neuropathy; sleep-onset transition; screening biomarker; risk stratification; wearable screening

Table S1. STARD 2015 Checklist

#	STARD Item	Location
Title / Abstract		
1	Identified as a diagnostic accuracy study using at least one measure of accuracy	Title ("candidate screening biomarker"), Abstract (AUROC, sensitivity, specificity, odds ratio)
2	Structured summary including design, methods, results, conclusions	Abstract
Introduction		
3	Scientific and clinical background; study rationale	Sections 1.1 – 1.4
4	Study objectives and hypotheses	Section 1.5
Methods		
5	Whether data collection was planned before (prospective) or after (retrospective) index test and reference standard	Section 2.1 (retrospective secondary analysis of public dataset)
6	Eligibility criteria	Figure 1 (participant selection flow)
7	Basis for identifying potentially eligible participants	Section 2.1 (T2DM inpatients from the Cheng et al. dataset)
8a	Sufficient detail of index test to allow replication	Sections 2.3 – 2.5 (NeuroKit2 HRV extraction, SDANN1 computation, non-parametric AUROC)
8b	Sufficient detail of reference standard to allow replication	Section 2.1 (binary DR labels from original clinical dataset)
9	Definition and rationale for test positivity cut-offs or result categories	Section 2.5 (Youden's J index); Table 3b (multi-threshold analysis)
10a	Whether clinical information was available to index test performers	Section 2.5 (AUROC computed on full sample; no train/test split)
10b	Whether clinical information was available to reference standard assessors	Section 2.1 (DR labels assigned by original study clinicians, independent of HRV)
11	Rationale for reference standard selection	Section 2.2 (clinical DR diagnosis from original dataset)
12a	Definition and rationale for index test result categories	Section 2.5 (continuous SDANN1 → binarized via Youden threshold; three-tier risk stratification)
12b	Definition and rationale for reference standard result categories	Section 2.1 (binary present/absent DR labels)
13a	Whether participants were consecutive, random, or convenience series	Section 2.1 (consecutive inpatients, July 2019 – January 2021)
13b	Study setting and locations	Section 2.1 (Suzhou Science & Technology Town Hospital)

#	STARD Item	Location
14	Sample size: how determined and whether planned before the study	Section 2.2 (sample size determined by available public dataset); Section 4.4, Limitation #1
15	Planned analysis methods including handling of indeterminate results, missing data, and reference standard variability	Sections 2.4–2.5 (artifact exclusion, statistical analysis framework)
Results		
16	Flow diagram of participants	Figure 1 (STARD flow diagram)
17	Baseline demographic and clinical characteristics of participants	Table 1
18	Distribution of severity of disease in patients	Section 2.2 (binary DR classification; severity staging not available in original dataset)
19	Cross-tabulation of index test results and reference standard results	Table 4a (2×2 contingency table); Figure 3 (ROC curve)
20	Any adverse events from performing the index test or reference standard	Not applicable (secondary analysis of existing ECG data)
21a	Estimates of diagnostic accuracy and their precision	Tables 2–4 (AUROC, sensitivity, specificity with 95% CI; OR with 95% CI)
21b	Any indeterminate results and how they were handled	Section 2.4 (two participants excluded for ECG artifacts)
22	Reproducibility and variability of the test, including inter-observer variability	Section 3.5 (SDANN2 segmentation robustness check)
Discussion		
23	Study limitations including potential sources of bias, statistical uncertainty, and generalizability	Section 4.4 (five limitations listed)
24	Intended use and clinical role of the index test	Sections 4.4, 5 (pre-screening triage tool; hypothesis-generating pilot study)
Other		
25	Registration number and registry name	Not applicable (secondary analysis, not a prospective diagnostic study)
26	Where the full study protocol can be accessed	Sections 2.4–2.7 (all analytical details in Methods; software versions listed)
27	Funding and Conflicts of Interest	Funding and Conflicts of Interest section

Table S2. DeLong test comparing SDANN1 with traditional HRV indices (N=39)

Comparison	$AUROC_1$	$AUROC_2$	Δ AUROC	DeLong z	p
SDANN1 vs RMSSD	0.731	0.688	0.042	0.45	0.655
SDANN1 vs SDNN	0.731	0.672	0.058	1.14	0.257

DeLong tests were non-significant, as expected given the limited statistical power at N=39 to resolve AUROC differences of this magnitude. Non-significance does not imply equivalence; the convergent pattern—three SDANN variants all significant (Table 2), all nine non-SDANN predictors non-significant—and the window-specificity of SDANN1's discriminative power (Table 6) provide stronger evidence than individual pairwise AUROC comparisons. Δ AUROC values were computed from exact (unrounded) DeLong estimates and may differ by ±0.001 from rounded values in Table 3.

Abbreviations: AUROC, area under the receiver operating characteristic curve; DR, diabetic retinopathy; HRV, heart rate variability; RMSSD, root mean square of successive differences; SDANN1, standard deviation of the averages of normal-to-normal intervals calculated using 1-min segments; SDNN, standard deviation of normal-to-normal intervals.

Table S3. Internal validation: bootstrap optimism-corrected AUROC and leave-one-out cross-validation

Metric	Value
Apparent AUROC	0.731
Mean optimism (2,000 bootstrap resamples)	0.004
Corrected AUROC	0.727
LOO-CV sensitivity (fold-specific Youden threshold)	54.5% (6/11)
LOO-CV specificity (fold-specific Youden threshold)	82.1% (23/28)
LOO-CV fold-specific thresholds	23.6–24.6 ms (median 24.6)
LOO-CV AUROC (logistic model)	0.653

Bootstrap optimism correction estimates overfitting bias by comparing bootstrap-sample apparent performance with original-sample performance. The minimal optimism (0.004) indicates that a single-predictor model (SDANN1) with 39 observations has very low overfitting risk, and the corrected AUROC (0.727) is close to the apparent estimate. The standard bootstrap optimism-correction procedure was used, not the .632/.632+ estimators; the Monte Carlo standard error of the optimism estimate with 2,000 resamples was approximately 0.002, confirming adequate resampling precision at N = 39. Leave-one-out cross-validation (LOO-CV), in which the Youden-optimal threshold was re-derived within each training fold and applied to the held-out participant, yielded a sensitivity of 54.5% and a specificity of 82.1%; fold-specific thresholds were highly stable (23.6–24.6 ms; median 24.6 ms), and the LOO-CV AUROC of the corresponding logistic model was 0.653. LOO-CV provides a more conservative estimate of out-of-sample performance, as it additionally accounts for threshold-selection variability.

Abbreviations: AUROC, area under the receiver operating characteristic curve; DR, diabetic retinopathy; SDANN1, standard deviation of the averages of normal-to-normal intervals calculated using 1-min segments.

Table S4. Firth penalized logistic regression — SDANN1 adjusted odds ratios (N=39, artifact-filtered)

Model	SDANN1 OR (per 1-SD)	95% CI	*p*	Other covariate OR (95% CI, p)
SDANN1 only	0.42	0.14–0.96	0.039	—
+ Age	0.38	0.11–0.94	0.034	Age 0.80 (0.36 – 1.68, p = 0.544)
+ Age + HbA1c	0.45a	0.12–1.11	0.086	Age 0.86 (0.22 – 2.97, p = 0.802); HbA1c 1.15 (0.40 – 3.48, p = 0.728)
+ Age + AHI	0.42	0.11–1.02	0.056	Age 0.79 (0.35 – 1.71, p = 0.558); AHI 1.19 (0.60 – 2.92, p = 0.628)
+ Age + HbA1c + AHI	0.48	0.12–1.17	0.114	Age 0.79 (0.19 – 2.83, p = 0.682); HbA1c 1.09 (0.37 – 3.41, p = 0.753); AHI 1.23 (0.57 – 4.15, p = 0.594)

ORs are reported per 1-SD increase in standardized covariates. Firth's penalty was used to reduce bias in maximum likelihood estimates with small samples. The SDANN1 point estimate (OR ≈ 0.38–0.45) remained stable across nested models, although wide CIs are expected with 11 events. The +HbA1c model is based on N=27 due to missing values, reducing statistical power. The + Age + AHI model is based on N = 36 (AHI available for 36 of 39 participants; 11 events; EPV ≈ 3.7) and the full model (+ Age + HbA1c + AHI) on N = 24 (9 events; EPV ≈ 2.2); AHI was not statistically associated with DR in either of these exploratory models. Given the low events-per-variable ratios, all multivariable estimates are exploratory and illustrative.

Abbreviations: CI, confidence interval; DR, diabetic retinopathy; HbA1c, glycated hemoglobin; OR, odds ratio; RR, interval between successive R waves; SD, standard deviation; SDANN1, standard deviation of the averages of normal-to-normal intervals calculated using 1-min segments.

Table S5. AHI-stratified analysis (N=36, artifact-filtered)

Group	n	DR+	Mean SDANN1 (ms)	Within-group AUROC
AHI < 21.2 (mild OSA)	18	6	48.9 ± 23.1	0.736
AHI ≥ 21.2 (moderate-to-severe OSA)	18	5	38.9 ± 31.7	0.754

AHI data were available for 36 of 39 participants. Median-split stratification (AHI median = 21.2 events/hour). Comparable AUROCs across strata (0.736 vs. 0.754) indicate that SDANN1's discriminative power for DR exists in both mild and moderate-to-severe OSA strata, rather than being driven by one group.The Spearman correlation between SDANN1 and AHI was −0.28 ($p = 0.09$), not reaching statistical significance, suggesting that the autonomic dynamics captured by SDANN are largely independent of OSA severity.

Abbreviations: AHI, apnea–hypopnea index; AUROC, area under the receiver operating characteristic curve; DR, diabetic retinopathy; DR+, diabetic retinopathy present; OSA, obstructive sleep apnea; RR, interval between successive R waves; SDANN1, standard deviation of the averages of normal-to-normal intervals calculated using 1-min segments.

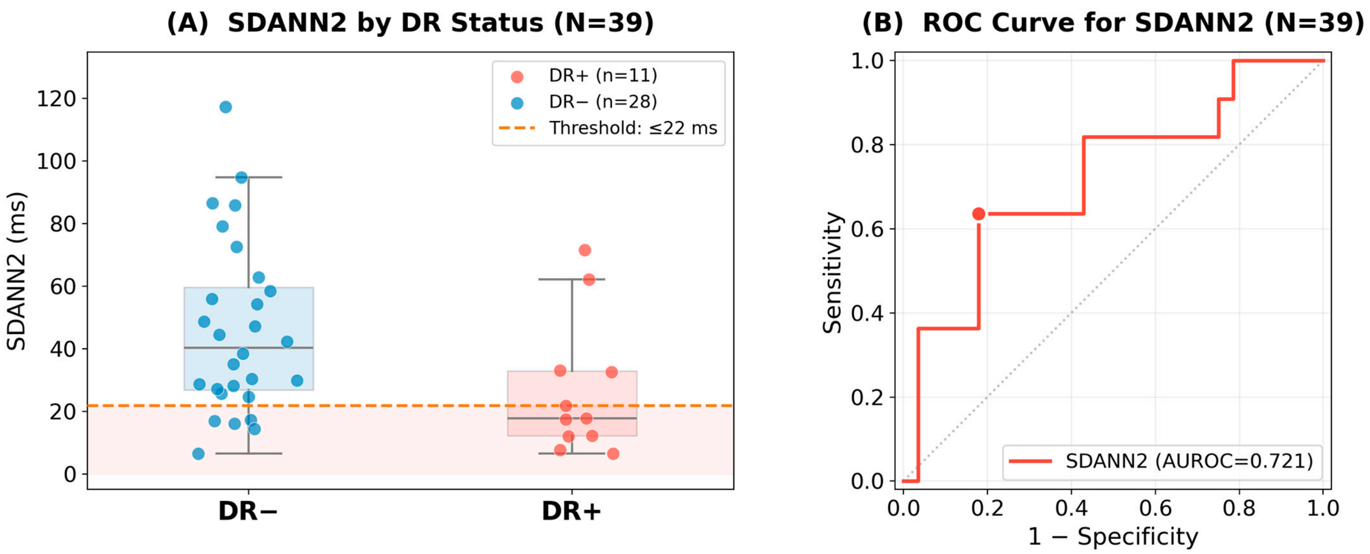

Figure S1. SDANN2 distribution and ROC curve (N=39)

(A) SDANN2 distribution by DR status. (B) SDANN2 ROC curve (AUROC = 0.721). SDANN2's discriminative performance is nearly identical to SDANN1, confirming segmentation robustness.

Abbreviations: AUROC, area under the receiver operating characteristic curve; DR, diabetic retinopathy; ROC, receiver operating characteristic; SDANN1, standard deviation of the averages of normal-to-normal intervals calculated using 1-min segments; SDANN2, standard deviation of the averages of normal-to-normal intervals calculated using 2-min segments.
